# Ultra-Smooth, Fully Solution-Processed Large-Area Transparent Conducting Electrodes for Organic Devices

**DOI:** 10.1038/srep36475

**Published:** 2016-11-03

**Authors:** Won-Yong Jin, Riski Titian Ginting, Keum-Jin Ko, Jae-Wook Kang

**Affiliations:** 1 Department of Flexible and Printable Electronics, Polymer Materials Fusion Research Center, Chonbuk National University, Jeonju 54896, Republic of Korea

## Abstract

A novel approach for the fabrication of ultra-smooth and highly bendable substrates consisting of metal grid-conducting polymers that are fully embedded into transparent substrates (ME-TCEs) was successfully demonstrated. The fully printed ME-TCEs exhibited ultra-smooth surfaces (surface roughness ~1.0 nm), were highly transparent (~90% transmittance at a wavelength of 550 nm), highly conductive (sheet resistance ~4 Ω ◻^−1^), and relatively stable under ambient air (retaining ~96% initial resistance up to 30 days). The ME-TCE substrates were used to fabricate flexible organic solar cells and organic light-emitting diodes exhibiting devices efficiencies comparable to devices fabricated on ITO/glass substrates. Additionally, the flexibility of the organic devices did not degrade their performance even after being bent to a bending radius of ~1 mm. Our findings suggest that ME-TCEs are a promising alternative to indium tin oxide and show potential for application toward large-area optoelectronic devices via fully printing processes.

For the past several years, motivation for developing flexible optoelectronic technologies has proceeded toward the achievement of fabricating low-cost plastic substrates, due to solution processing, low-cost materials, and lightweight devices^1^,^2^. Presently, the most commonly used transparent electrodes in optoelectronics devices have been indium tin oxides (ITO). However, several drawbacks exist with regard to their usage, i.e., high production costs due to vacuum deposition requirements, rapidly depleting indium sources, and poor mechanical properties resulting in crack formation under low bending stress values^3^. Accordingly, the realization of mechanically stable large-area optoelectronics devices on flexible substrates would require the development of alternative transparent conducting electrode (TCE) materials to replace ITO. Thereby, finding suitable ITO replacements remains a challenge.

Extensive effort has been devoted to the replacement of ITO with alternative solution-processed materials for flexible TCEs such as graphene^4^,^5^, poly(3,4-ethylenedioxythiophene) polystyrenesulfonate (PEDOT:PSS)^6^,^7^, carbon nanotubes^8^,^9^, and metal oxides^10^,^11^. Despite their potential as an ITO replacement, these materials suffer from classic trade-offs between optical transmittance and electrical conductivity. Thicker layers afford high conductivity, but this increase comes at the expense of optical transmittance and vice versa, thus frequently affecting the performance of optoelectronic devices^12^. It is possible to improve the conductivity of TCEs through the use of metallic materials such as metal nanowires (NWs) and metal nano-mesh/micro-mesh materials^13^,^14^. Recently, metal NWs, such as silver NWs, copper NWs, and gold NWs, have shown promise as alternative TCEs due to their high conductivities, transparency, and excellent flexibility^15^,^16^,^17^. However, metal NW-based TCEs typically possess high surface roughness due to interconnections between their junctions; the materials are also easily oxidized in air, often degrading the performance of optoelectronic devices^18^,^19^. Metal micro- and nano-mesh electrodes have attracted considerable attention recently because the thickness, spacing, and line-widths of metal patterns can be easily modified to obtain desirable optical and electrical properties with the benefit of air-processable conditions. These metal meshes have been fabricated by various methods such as pattern-masked evaporation^20^,^21^, nanoimprint lithography^17^,^22^,^23^, inkjet^24^, flexographic^25^, transfer^26^,^27^ and gravure-offset printing^12^,^28^. However, these electrodes also suffer from high surface roughness, resulting in the possibility of electrical short-circuits between the TCEs and the top electrode. To prevent this, metallic TCEs should be embedded within the polymer substrate^29^. These techniques have shown TCEs to be promising for large-area flexible substrates with the advantages of thicker metal grids that can provide additional conductive current paths^30^. Previously, we have demonstrated flexible TCEs comprised of Ag grids with a combination of vacuum-sputtered amorphous-ITO in organic solar cells (OSCs) and organic light-emitting diode (OLED) applications^12^. Although promising results for the optical, electrical, and mechanical properties have been demonstrated, the device performance of OSCs and OLEDs is still relatively low compared to ITO/glass-based devices, and still requires vacuum-deposited ITO electrodes.

Herein, we report fully printed TCEs under air atmosphere conditions with high conductivities (sheet resistance ~4 Ω □^−1^), high transparency (optical transmittance ~90% at a wavelength of 550 nm), low roughness (root-mean-square roughness ~1 nm), and high flexibility (bending radius ~1 mm) through the simultaneous use of a combination of embedded metal grids and conducting polymers within a polymer substrate. Henceforth, these materials are denoted as metal grid-embedded TCEs (ME-TCEs). ME-TCEs were used to fabricate flexible OSCs and OLEDs that exhibited performance values that were close to and superior to those of devices fabricated on ITO/glass substrates, respectively. The flexibility of flexible ME-TCEs and organic devices did not reveal degradation in their performance even after demonstrating a bending radius of ~1 mm. Our findings suggest that ME-TCEs are promising alternatives to ITO with potential applications in large area optoelectronic devices.

## Experimental Procedures

### Fabrication of the bendable substrates

**Figure 1:**
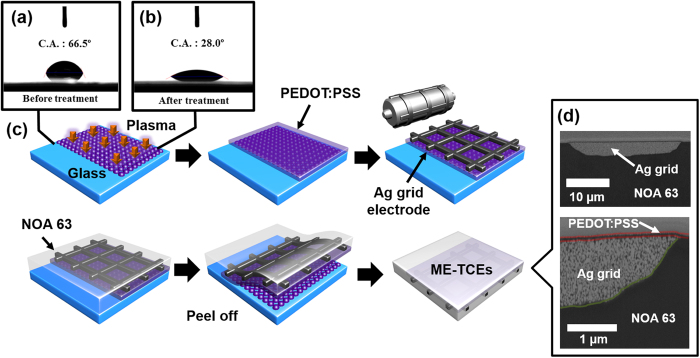
Schematic novel fabrication process of the ME-TCEs film. Photographs of the measured contact angle of: (**a**) bare glass and (**b**) N_2_ plasma treated glass. (**c**) Schematic illustration for the fabrication of full solution and air processed ME-TCEs. (**d**) Cross-sectional SEM images of a PEDOT:PSS/Ag grid embedded within the polymer substrate (*i.e*., ME-TCEs).

First, glass substrates (Corning, Co. Korea) were each successively ultrasonicated in acetone, ethanol, and boiled isopropyl alcohol (IPA) for 5 mins. Subsequently, the substrates were dried at 120 °C for 30 mins in a laboratory oven. Surface treatment was performed with a commercially available plasma treatment system (GENIA Tech) with a radio frequency (RF) power density of 1.6 mW/cm^2^ at a pressure of 0.2 Torr for 15 s with a controlled N_2_ gas flow of 80 sccm, generating a hydrophilic surface, as shown in Fig. 1. A PEDOT: PSS (Clevious P PH 1000) layer was spin coated at 2000 rpm for 40 s onto the plasma-treated glass substrates (150 mm × 150 mm) followed by annealing at 150 °C for 5 min; the surfaces were additionally treated with ethylene glycol (Aldrich, 99.8%) to improve their conductivity and were annealed at 150 °C for 5 mins in ambient air, yielding thicknesses of ~90 nm. Ag grids with an approximate thickness of ~1.3 μm and width of ~32 μm were measured via surface profilometry (KLA Tencor, P-10) and were printed with a Ag ink paste (70.82% Ag content, Advanced Nano Product Co.) via a gravure off-set method (DCN Co, Ltd., Roll printer) onto the PEDOT:PSS layer; this was followed by thermal curing at 200 °C for 1 hr in a laboratory oven. Meanwhile, the sheet resistance of PEDOT:PSS only slightly increases upon annealing at 200 °C in an oven as shown in Figure S1. The UV-curable polymer NOA 63 (Norland Products Inc.) was spin coated onto the PEDOT:PSS/Ag grid composite layer at 1000 rpm for 40 s and was cured under UV light (365 nm, 20 mWcm^−2^, NEXTRON, LIT-2000) for 20 mins, yielding a layer thickness of ~50 μm. Finally, the PEDOT:PSS/Ag grid embedded-transparent conducting electrodes (*i.e*., ME-TCEs) were detached from the glass substrates and cut into various sample sizes using a cutter or another sharp utensil (A video of the fabrication process is available as Supporting Video).

### Fabrication of the bendable inverted OSCs

Prior to spin coating the ZnO layer, the ME-TCE films were subjected to a N_2_ plasma treatment at 10 W and 0.2 Torr for 30 s. The ZnO precursor was first prepared by dissolving zinc acetate dihydrate (Zn(CH_3_COO)_2_·2H_2_O, Aldrich, 99.9%, 1.64 g) and ethanolamine (NH_2_CH_2_CH_2_OH, Aldrich, 99.5%, 0.5 g) in 2-methoxyethanol (CH_3_OCH_2_CH_2_OH, Aldrich, 99.8%, 10 g)^31^,^32^ under vigorous stirring for 30 mins without heat in a hydrolysis reaction. The ZnO precursor was then filtered with a 0.25 μm polypropylene (PP) filter and spin-coated onto the substrates at 5000 rpm for 40 s, followed by annealing at 150 °C for 20 min. A mixed solution of PTB7 and PC_71_BM (1:1.5) in chlorobenzene/1,8-diiodoctane (DIO) (97:3 by volume) was stirred at 60 °C overnight and filtered through a 0.20 μm polytetrafluoroethylene (PTFE) filter. The ZnO coated substrates were then transferred to a nitrogen glove box and the active layer solution was spin-coated at 1000 rpm for 40 s to yield a layer thickness of approximately 90 nm. Subsequently, PEDOT:PSS (Clevios P VP AI 4083) in isopropyl alcohol at a ratio of 1:15 was spin-coated onto the active layer to obtain a film with a thickness of approximately 10 nm. The devices were subsequently transferred to a vacuum chamber without further annealing. Finally, an Ag top electrode was deposited through a shadow mask depending on the device active area (rate of 0.2 nm s^−1^) via thermal evaporation in a vacuum pressure of 10^−6 ^Torr.

### Fabrication of bendable OLEDs

The phosphorescent emission layer (EML) consisted of a blend of poly(N-vinyl carbazole) (PVK), N,N′-Bis(3-methylphenyl)-N,N′-diphenyl-[1,1′-biphenyl]-4,4′-diamine) (TPD), 2-(tert-butylphenyl)-5-biphenylyl-1,3,4-oxadiazole) (PBD), and 2-(4-biphenyllyl)-5-(4-tert-butylphenyllyl)-1,3,4-oxadiazol (Ir(mppy)_3_) in chlorobenzene with a blend ratio of 0.61, 0.09, 0.24, and 0.06 mg/ml, respectively. PVK (Mw = 1,100,000) was purchased from Aldrich, whereas, TPD, PBD, and Ir(mppy)_3_ were purchased from Electronic Materials Index. To fabricate the flexible OLEDs, PEDOT:PSS (Clevios PVP A 4083) was filtered and spin-coated onto ME-TCEs substrate to yield a 30 nm layer and were annealed at 150 °C for 5 mins in air. A 44 nm thick EML was achieved by spin coating the solution blend of PVK:TPD:PBD:Ir(mppy)_3_ in chlorobenzene onto the PEDOT:PSS layer and annealing at 80 °C for 20 mins under an atmosphere of N_2_. The electron-transport layers (ETL), 2,2′,2′-(1,3,4-phenylene)-tris[1-phenyl-1H-benzimidazole] (TPBI, 40 nm) (Nichem Fine Technology Co., Ltd.) were deposited on top of the EML via thermal evaporation. Subsequently, lithium fluoride (LiF, 1 nm) and aluminum (Al, 100 nm) were deposited via thermal evaporation at a base pressure of 10^−6 ^Torr. The emission area of the devices was 0.11 cm^2^.

### Characterization

The surface morphology and cross-sections of the ME-TCEs were recorded via field-emission scanning electron microscopy (FESEM, Helios 650) equipped with a focused ion beam. The surface topography of the substrates was characterized via atomic force microscopy (AFM, Park systems, NX-10 microscope) in semi-contact mode. In order to accurately measure the sheet resistance of the ME-TCE substrates, a non-contact resistance measurement instrument (Napson, EC-80 P) was employed. The photovoltaic characteristics of the OSC devices were measured in ambient air under AM 1.5G illumination at 100 mWcm^−2^ and the current density–voltage (*J–V*) curves were measured with a Keithley 2400 SMU. The external quantum efficiency was measured using an EQE system (Oriel IQE-200), which was calibrated with a silicon photodetector. Film thicknesses were measured with an Alpha-Step, and the current density-voltage-luminance characteristics of the devices were measured with a Keithley 236, meanwhile the electroluminescence (EL) spectra and international commission on illumination (CIE) coordinates were obtained using a spectrophotometer (Photo-research, CS-2000).

## Results and Discussion

Figure 1 schematically demonstrates the main step-by-step process with regard to fabricating solution-processed, highly transparent, highly conductive, and extremely smooth ME-TCEs under an air atmosphere. Details with regard to the fabrication conditions are explained in the experimental section. A key concept of the fabrication process is a fully embedded architecture consisting of both the metal-grid and conducting polymer within the flexible substrate, leading to extremely smooth surface morphology. Figure 1(a,b) depicts the N_2_ plasma treatment that was employed to improve the wetting properties of the glass substrate, where the contact angle of PEDOT:PSS solution on top of glass surface was significantly suppressed from 66.5 to 28.0°, rendering it hydrophilic. These steps are important for the ease of ME-TCEs film detachment from the glass substrate. The Ag grids were printed onto the PEDOT:PSS-coated glass with different spacing sizes (4, 2 and 1 mm) or denoted as grid pitch (GP); the detailed process can be refer to Experimental Procedures. Subsequently, UV-curable NOA 63 polymer was spin-coated onto the Ag-grid/PEDOT:PSS/glass substrate, resulting in a layer thickness of 50 μm, as shown in Fig. 1(c). Finally, the film was cut to the desired size and detached from the glass substrate resulting in PEDOT:PSS/Ag grids embedded within flexible substrates, i.e., ME-TCEs. Cross-sectional scanning electron microscopy (SEM) images of the ME-TCE substrates were obtained by using a focused ion beam as shown in Fig. 1(d). It was clearly observed that both the printed Ag grids and PEDOT:PSS were fully embedded within the polymer substrate.

**Figure 2:**
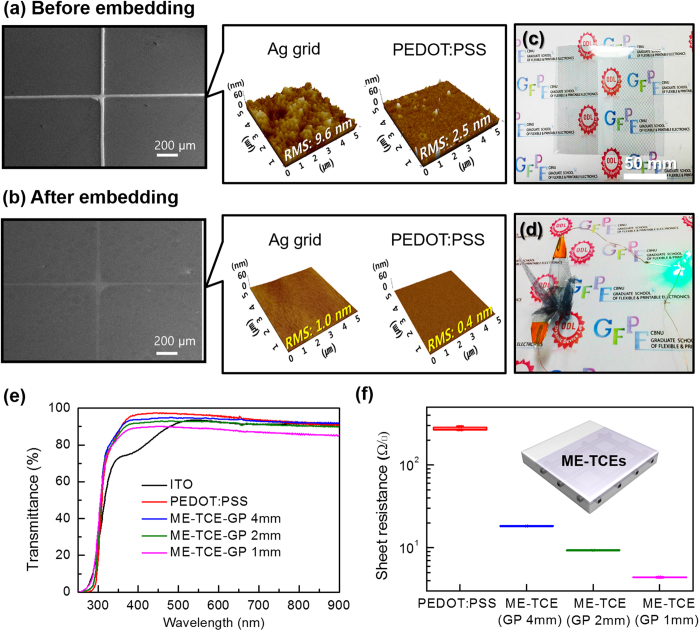
FESEM top-view and AFM topography images (**a**) prior to embedding and (**b**) after embedding the ME-TCEs film. (**c**) Photograph of the flat ME-TCEs (20 cm × 20 cm) film substrate and (**d**) blue LEDs connected to an ME-TCEs film that was folded into an origami bird shape. (**e**) Transmittance spectra and (**f**) sheet resistance as a function of GP size for the ME-TCEs films.

Figure 2(a,b) show the surface of the ME-TCE films after embedding, which showed root-mean-square (rms) surface roughness <1.0 nm and maximum peak-to-valley height <7 nm, dramatic reductions compared to the surface before embedment (rms roughness <10 nm and peak-to-valley height <50 nm). These findings show that the fully solution-processed ME-TCE films exhibited extremely smooth surfaces. Additionally, the photograph exhibits high transparency with a symbol mark in the background that could be clearly seen through the ME-TCE substrate as seen in Fig. 2(c); the film also exhibited excellent mechanical flexibility, demonstrating functional blue-light emission of the LED even after ME-TCE, with the ability to be folded into an origami bird shape (Fig. 2(d)). As a result, the fabricated ME-TCEs demonstrate great potential for applications in various flexible optoelectronic devices. Figure 2(e) depicts the transmittance spectra of the ME-TCE films with various GP sizes. For comparison, ITO exhibited a transmittance value of ~94% at a wavelength of 550 nm (excluding the substrate), whereas pristine PEDOT:PSS (without a Ag grid) exhibited a transmittance value of ~96%. By inserting Ag grids with different GP sizes, the transmittance values decreased slightly from ~95% (ME-TCEs-GP 4 mm) to ~90% (ME-TCEs-GP 1 mm). Accordingly, the pristine PEDOT:PSS films exhibited sheet resistance (*R*_*sheet*_) values of ~250 Ω □^−1^ and decreased significantly to ~4 Ω □^−1^ after inserting the Ag grids (ME-TCEs-GP 1 mm) as shown in Fig. 2(f).

**Figure 3:**
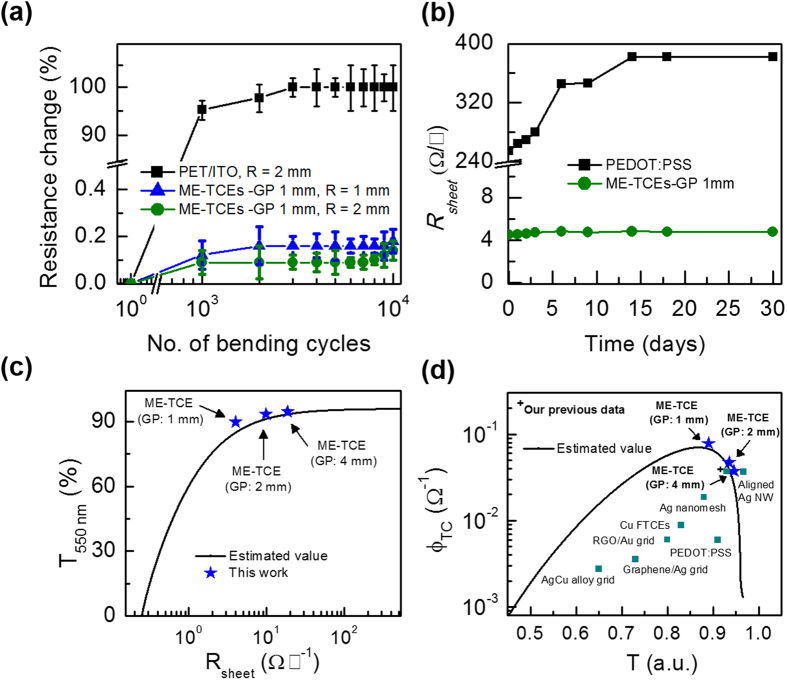
(**a**) Resistance changes of the TCEs with various quantities of bending cycles and bending radii. (**b**) Sheet resistance (*R*_*sheet*_) values of TCEs films stored in ambient air at 25 °C vs. storage time. (**c**) Optical transmittance (*T*_*550 nm*_) values plotted as a function of sheet resistance for ME-TCEs with different GP sizes. (**d**) Φ_TC_ values as a function of transmittance. For comparison, the Φ_TC_ value of Ag mesh^45^, aligned Ag NW^15^, Ag nanomesh^26^, Cu flexible TCEs (FTCEs)^46^, reduced graphene oxide (RGO)/Au grids^4^, graphene/Ag grids^47^, AgCu alloy mesh^48^, and modified PEDOT:PSS^6^ are also shown.

The mechanical bending properties of the ME-TCE films with different bending radii and bending cycles are shown in Fig. 3(a), which are based on the average values of four different batches of samples. The percentage changes in resistance of the flexible electrodes can be denoted as the ratio between the change in resistance after bending and the initial resistance. Interestingly, the ME-TCEs-GP 1 mm exhibited superior mechanical flexibility and excellent fatigue strength with a change in resistance of less than 0.2% amid being bent 10,000 times at a bending radius of 1 mm. In comparison to the ITO/PET films, the resistance change by more than 10-fold and it is most likely that the ITO layer was cracked due to its poor mechanical properties. Additionally, reliability of the ME-TCEs was determined by monitoring the changes of *R*_*sheet*_ during long-term storage in ambient air at 25 °C (RH ~30%) up to 30 days as shown in Fig. 3(b). The ME-TCE films were considerably stable in air without significant changes to *R*_*sheet*_. On the contrary, PEDOT:PSS embedded within the NOA63 substrate exhibited a gradual increase in *R*_*sheet*_ from 250 Ω □^−1^ up to 380 Ω □^−1^. The significant increase of *R*_*sheet*_ in air was expected due to the acidic nature of PEDOT:PSS and its chemical instability due to the moisture in air^33^,^34^,^35^. Interestingly, the degradation of PEDOT:PSS did not significantly affect the Ag grid underneath (96% of its initial resistance was retained up to 30 days), which was unlike the corrosion that was widely reported with ITO^36^. In other words, the Ag grid is highly stable in ambient air and chemically stable toward PEDOT:PSS degradation. It is noteworthy to mention that the changes in *R*_*sheet*_ of PEDOT:PSS did not affect the total *R*_*sheet*_ as clearly seen in Figures S2(a,b), due to the parallel connection between PEDOT:PSS and the Ag grid. Therefore, the major contribution to the total *R*_*sheet*_ is from the resistance of the Ag grid itself.

Figure 3(c) demonstrates the relationship between the transmittance of the ME-TCE film at a wavelength of 550 nm (*T*_*550 nm*_) as a function of *R*_*sheet*_, where the estimated value was calculated based on the aperture ratio of the metal grid^12^. In general, the performance of a transparent conductor can be determined by figure of merit (FoM) values of * σ*_*DC*_/*σ*_*Opt*_ (λ at 550 nm), as shown in Eq. 1, where *σ*_*DC*_ and *σ*_*Opt*_ represent the direct current and optical conductivity, respectively^37^.


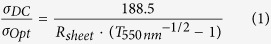


This expression has been widely used to describe the quality and performance of transparent conducting films, where typical FoM values greater than 35 are a minimum requirement for the commercial viability of transparent electrodes^38^. In the present work, the FoM value of *σ*_*DC*_/*σ*_*Opt*_ increased from 350 (GP 4 mm) to 790 (GP 1 mm), which is significantly two-fold higher than that of earlier studies based on a-ITO/Ag-grids (FoM ~392)^12^ and PET/ITO (FoM ~200)^39^. Notably, these values are extremely large for transparent electrodes based on metal-grids. Therefore, these methods can be used to overcome the drawbacks between transmittance and conductivity, where PEDOT:PSS/Ag-grid embedded polymer substrates demonstrate high conductivity while maintaining high transparency. Another FoM can be defined as 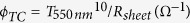 to determine the practical limitation of ME-TCEs with different GPs^40^, and the estimated values of T_550 nm_ and R_sheet_ were simulated based on different aperture ratio of the Ag grid. As shown in Fig. 3(d), with decreasing GP sizes in ME-TCE films up to 1 mm, higher *ϕ*_*TC*_ values can be reached. The optimum *ϕ*_*TC*_ value of 85 × 10^−3 ^Ω^−1^ with ME-TCEs-GP 1 mm films was much higher than that of 18 × 10^−3^ and 36 × 10^−3 ^Ω^−1^ for Ag-mesh^26^ and aligned Ag NWs^15^, respectively, as well as other TCEs. Additionally, the estimated value showed that further decreases to the optimum GP size below 1 mm would suffer from low transmittance and a significant drop in the *ϕ*_*TC*_ value.

**Figure 4:**
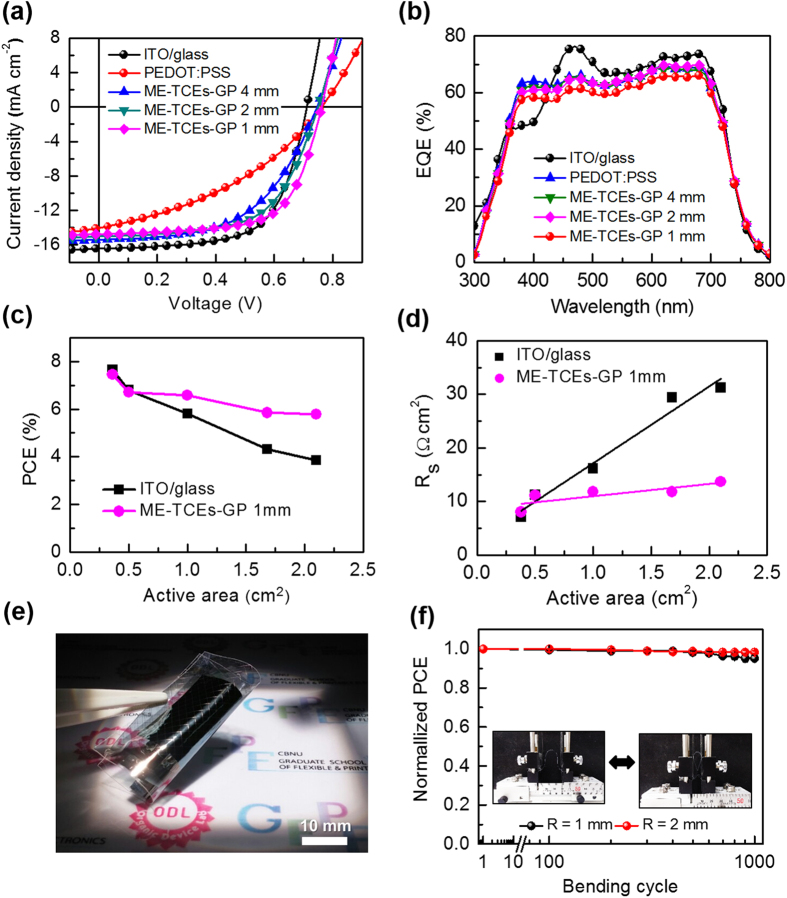
(**a**) *J-V* characteristics and (**b**) IPCE spectra of the IOSCs fabricated with various ME-TCEs GP sizes with an active area of 0.38 cm^2^. (**c**) The measured PCE value as a function of device active area. (**d**) The measured series resistance values as a function active areas. (**e**) Photography image of flexible IOSC device based on ME-TCEs GP 1 mm with device active area 2.10 cm^2^. (**f**) The measured PCE of the flexible IOSCs as a function of bending cycles at a radius of 1 and 2 mm, normalized to the initial value.

To investigate the potential of the ME-TCEs as a transparent electrode, an inverted OSC (IOSC) devices with a device structure of ME-TCEs/ZnO/photoactive-layer/PEDOT:PSS/Ag was fabricated. The photoactive layer was a bulk heterojunction blend consisting of thieno[3,4-b]thiophene/benzodithiophene (PTB7) and [6,6]-phenyl C71 butyric acid methyl ester (PC_71_BM). Various devices with different GP sizes were fabricated and subsequently ITO/glass and PEDOT:PSS-only were used for comparison. Figure 4(a) shows the current density-voltage (*J-V*) characteristics of the IOSCs based on ME-TCE films which were measured under AM 1.5 G illumination with a device active area of 0.38 cm^2^. Additionally, the average values of four samples with extracted photovoltaic parameters can be seen in Table 1. The reference device with an ITO-coated glass substrate exhibited a short-circuit current density (*J*_*sc*_) of 16.67 mA cm^−2^, open circuit voltage (*V*_*oc*_) of 0.710 V, and fill-factor (FF) of 64.6%, resulting in a power conversion efficiency (PCE) of 7.65%. Remarkably, the IOSCs fabricated using ME-TCEs-GP 1 mm films exhibited high PCEs of 7.46%, amid a slightly reduced *J*_*sc*_ of 14.93 mA cm^−2^; however, the efficiency was compensated by an improvement in the FF up to 68.5% and the *V*_*oc*_ up to 0.729 V. An enhancement of the FF could be explained due to the decrease in *R*_*sheet*_, and the increase in *V*_*oc*_ could be attributed to a reduced leakage current (as shown in Figure S3). Meanwhile, further increasing the GP size (ME-TCEs-GP 4 mm), the *J*_*sc*_ increased even more to 15.30 mA cm^−2^, however, FF was lowered due to higher *R*_*sheet*_ of the ME-TCEs. As a result, the PCE decreased noticeably to 6.18%. By contrast, the PEDOT:PSS film (without Ag grid) exhibited an even lower PCE of 3.50% due to a considerably low FF value. Additionally, the above findings were in good agreement with the estimated value of *ϕ*_*TC*_, which yielded the optimum value in the ME-TCEs-GP 1 mm film. Figure 4(b) depicts the EQE spectra for all the devices. The calculated *J*_*sc*_ from the EQE spectra exhibited a similar trend in the *J*_*sc*_ value obtained from the *J-V* measurement. Furthermore, there was a correlation between the ME-TCE GP size and *J*_*sc*_ value, where higher GP sizes yielded higher *J*_*sc*_ values. This negative effect could be explained by shadow loss from the Ag grid electrode resulting in a decreased *J*_*sc*_ value in the device, which was commonly observed in IOSC devices, where shadow losses increased linearly with the number of grid lines^41^,^42^,^43^.

Figure 4(c) depicts the PCE as a function of active area for the ITO and ME-TCE based IOSCs. It was clearly observed that IOSCs based on ITO substrates yielded a linear decrease in PCEs from 7.65% to 3.85% for 0.38 cm^2^ and 2.10 cm^2^, respectively, resulting from a drastic FF reduction from 64.6% to 36.9% (as shown in Figure S4 and Table S1). Meanwhile, ME-TCEs-GP 1 mm film based devices exhibited relatively high PCEs of 5.79% up to an active area of 2.10 cm^2^ due to a small FF drop from 68.5% to 60.0% (as summarized in Table S1). The photography images of large device active area of 2.10 cm^2^ for flexible IOSC device based on ME-TCEs-GP 1 mm substrate are depicts in Fig. 4(e). Furthermore, for the ITO based substrates, a linearly increasing trend of series resistance (*R*_*s*_) was observed with increases in device active area, where a linear slope of 14.3 Ω was recorded as shown in Fig. 4(d)^44^. Remarkably, the IOSC devices based on ME-TCE substrates produced only slight changes in the *R*_*s*_ values with a slope of 2.28 Ω, which was 6-fold lower than that of ITO/glass. It has been reported that the slope originated from the combination of *R*_*sheet*_ and the cell geometry factor^34^. Based on this result, it showed that the ME-TCE films have potential to be applied in large area optoelectronics. Furthermore, the mechanical stability of the flexible IOSCs was measured at the low bending radii (*R*) of 1 and 2 mm, as shown in Fig. 4(f), and its bending radii dependence can be seen in Figure S5. The flexible IOSCs based on ME-TCEs retained PCE values up to ~95% and ~98% even after 1,000 bending cycles for *R* ~1 mm and 2 mm, respectively.

**Figure 5:**
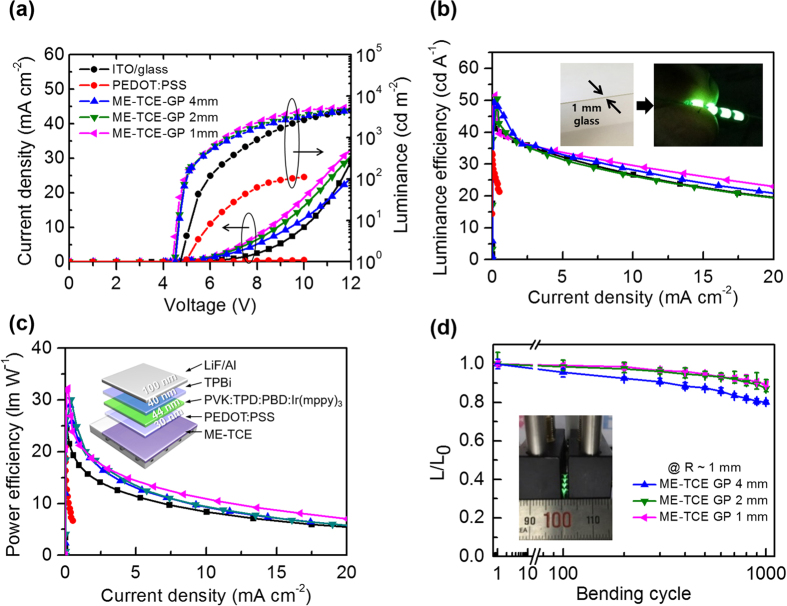
(**a**) Current density-voltage-luminance characteristics of the OLEDs fabricated with different TCEs. (**b**) Luminance efficiency-current density characteristics of the OLEDs (inset shows photographs of the bendable OLEDs). (**c**) Power efficiency-current density characteristics of the OLEDs (inset shows a schematic of the flexible OLEDs). (**d**) The measured intensity of light emitted by the flexible OLEDs as a function of bending cycles at different bending radii, which were normalized to the initial value.

OLEDs performance has been reported as being highly dependent on surface roughness, where high surface roughness could result in shunted pathways, resulting in lower device performance. Therefore, the performance of OLEDs based on ME-TCEs was beneficial due to its extremely low surface roughness, preventing leakage and shunt currents in the devices. In order to further evaluate the performance and compatibility of the ME-TCEs, flexible OLEDs were fabricated using the following device architecture: ME-TCEs/PVK:TPD:PBD:Ir(mppy)_3_/TPBI/LiF/Al, where PVK is poly(N-vinyl carbazole), TPD is N,N′-Bis(3-methylphenyl)-N,N′-diphenyl-[1,1′-biphenyl]-4,4′-diamine), PBD is 2-(tert-butylphenyl)-5-biphenylyl-1,3,4-oxadiazole), Ir(mppy)_3_ is 2-(4-biphenyllyl)-5-(4-tert-butylphenyllyl)-1,3,4-oxadiazol, and TPBI is 2,2′,2′-(1,3,4-phenylene)-tris[1-phenyl-1H-benzimidazole]. The characteristic properties of these devices can be seen in Fig. 5 and are summarized in Table 2; comparisons are made with a control device fabricated onto ITO-coated glass substrates (Fig. 5(a–c)). Accordingly, as observed in Fig. 5(a), ME-TCE based OLEDs exhibited smaller leakage currents with a turn-on voltage of 4.2 V compared to ITO/glass and PEDOT:PSS substrates, which could be attributed due to extremely smooth surfaces (as shown in Figure S6). The luminance efficiency (LE) and power efficiency (PE) of the devices can be seen in Fig. 5(b,c) as a function of current density. The device with ME-TCEs-GP 1 mm films exhibited a maximum LE of 50.2 cd A^−1^ and PE of 32.2 lm W^−1^, which was higher than that of the ITO/glass (LE ~42.8 cd A^−1^ and PE ~26.0 lm W^−1^) and PEDOT:PSS (LE ~33.3 cd A^−1^ and PE ~20.9 lm W^−1^). The ME-TCE devices also exhibited excellent mechanical flexibility, as seen in Fig. 5(d), with only a small decrease in the emitted light intensity.

## Conclusion

In conclusion, highly bendable organic devices such as IOSCs and OLEDs were fabricated using ultra-smooth and fully printed ME-TCE substrates. The use of a fully embedded architecture that simultaneously incorporated both a metal-grid and conducting polymer into a flexible substrate, led to an extremely smooth surface and an increase in conductivity without a reduction in transmission. This study provided insight toward overcoming the classic trade-off between optical transmittance and electrical conductivity via facile fabrication methods. The flexibility of the organic devices did not degrade their performance even after being bent to a bending radius of ~1 mm. Our findings suggest that ME-TCEs are a promising alternative to ITO and show potential for application toward large-area optoelectronic devices via solution based printing processes.

## Additional Information

**How to cite this article:** Jin, W.-Y. *et al*. Ultra-Smooth, Fully Solution-Processed Large-Area Transparent Conducting Electrodes for Organic Devices. *Sci. Rep.* **6**, 36475; doi: 10.1038/srep36475 (2016).

**Publisher’s note:** Springer Nature remains neutral with regard to jurisdictional claims in published maps and institutional affiliations.

## Supplementary Material

Supplementary Material

Supplementary Movie

## Figures and Tables

**Table 1:** Photovoltaic characteristics of flexible IOSCs based on ME-TCEs with different GP sizes.

Device	Transmittance (%)	Sheet resistance (Ω ◻^−1^)	*V*_*oc*_ (V)	*J*_*sc*_ (mA/cm^2^)	FF (%)	PCE (%)
ITO/glass	93.6	10	0.710 ± 0.001	16.67 ± 0.07	64.62 ± 0.10	7.65 ± 0.01
PEDOT:PSS	96.6	250	0.720 ± 0.001	14.26 ± 0.08	36.46 ± 0.05	3.50 ± 0.09
ME-TCEs-GP 4 mm	94.6	19	0.722 ± 0.001	15.30 ± 0.15	55.98 ± 0.80	6.18 ± 0.16
ME-TCEs-GP 2 mm	93.5	9	0.732 ± 0.009	15.03 ± 0.09	61.79 ± 0.76	6.80 ± 0.17
ME-TCEs-GP 1 mm	89.8	4	0.729 ± 0.001	14.93 ± 0.13	68.50 ± 0.01	7.46 ± 0.07

**Table 2:** Summary of flexible OLED performance based on ME-TCEs with different GP sizes.

Device	Turn-on^a)^ (V)	Maximum LE^b)^ (cd A^−1^)	Maximum PE^c)^ (lm W^−1^)	@1000 cd m^−2^	
				LE (cd A^−1^)	PE (lm W^−1^)
ITO/glass	4.5	42.87 ± 2.31	26.08 ± 1.85	34.01 ± 0.73	12.57 ± 1.14
PEDOT:PSS	4.9	33.38 ± 1.28	20.97 ± 1.89	—	—
ME-TCEs-GP 4 mm	4.2	47.73 ± 1.62	29.31 ± 3.76	35.70 ± 3.00	15.28 ± 1.81
ME-TCEs-GP 2 mm	4.2	50.18 ± 1.36	31.26 ± 1.77	35.63 ± 3.19	15.97 ± 2.03
ME-TCEs-GP 1 mm	4.2	50.27 ± 1.74	32.23 ± 1.85	35.94 ± 0.32	16.85 ± 0.15

^a)^Turn on voltage with luminance of 1 cd m^−2^; ^b)^Maximum LE: maximum luminance efficiency; ^c)^Maximum PE: maximum power efficiency.
