# Supplementary material for: Ultra-Smooth, Fully Solution-Processed Large-Area Transparent Conducting Electrodes for Organic Devices
Source: Sci Rep. 2016 Nov 3;6:36475. doi: 10.1038/srep36475 (PMC5093558; doi:10.1038/srep36475)
Supplement: Supplementary Material [file srep36475-s1.doc]

Supplementary Information

**Ultra-Smooth, Fully Solution-Processed Large-Area Transparent Conducting Electrodes for Organic Devices**

Won-Yong Jin^1^, Riski Titian Ginting^1^, Keum-Jin Ko^1^, and Jae-Wook Kang^*^

Department of Flexible and Printable Electronics, Polymer Materials Fusion Research Center, Chonbuk National University, Jeonju 54896, Republic of Korea

^1^These authors contributed equally to this work.

*To whom correspondence should be addressed.

Jae-Wook Kang (E-mail: [jwkang@jbnu.ac.kr](mailto:jwkang@jbnu.ac.kr))

Figure S1 depicts the *R_sheet_* of PEDOT:PSS as function of temperature both in air for 5 min and laboratory oven for 1 h each. The R_sheet_ shows small changes when annealed at 150 ^o^C for 1 h in oven as compared with annealing in air for 5 min and slightly increased with annealing temperature. Our findings is in good agreement with previous report, which claimed that material mass and chemical properties of PEDOT:PSS does not show any significant changes below than 250 ^o^C.

**Figure S1.** *R_sheet_* of PEDOT:PSS as function of temperature in air for 5 minute and 1 h in the laboratory oven.


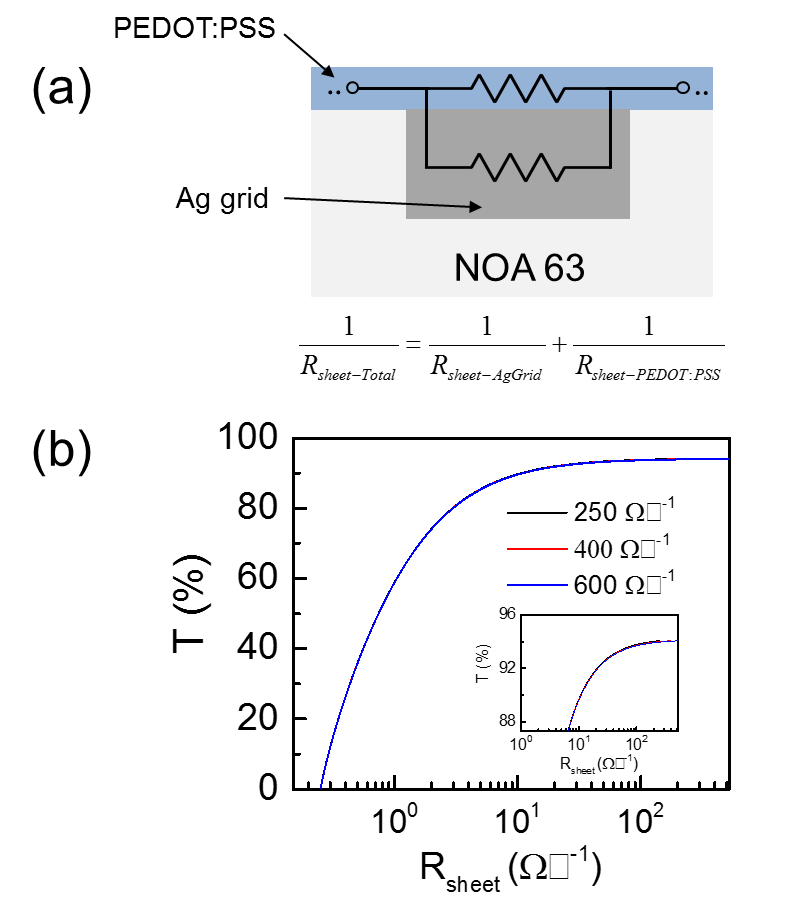


**Figure S2.** (a) Schematic diagram of PEDOT:PSS/Ag-grid embedded into NOA 63 with the respective equation for total *R_sheet_*, (b)Simulation of transmittance T_550_ _nm_ as function of *R_sheet_* with different *R_sheet_* of PEDOT:PSS varied from 250 to 600 Ω □^-1^.

**Table S1.** Photovoltaic parameters of ITO and ME-TCEs based IOSCs with various active areas.

| Substrate | Area  (cm^2^) | V_oc_  (V) | J_sc_  (mA cm^-2^) | FF  (%) | PCE  (%) |
| --- | --- | --- | --- | --- | --- |
| ITO/Glass | 0.38 | 0.710 | 16.67 | 64.62 | 7.65 |
|  | 0.50 | 0.740 | 15.20 | 60.42 | 6.80 |
|  | 1.00 | 0.742 | 14.79 | 52.93 | 5.81 |
|  | 1.68 | 0.717 | 15.04 | 40.11 | 4.32 |
|  | 2.10 | 0.714 | 14.62 | 36.93 | 3.85 |
| ME-TCEs-GP 1mm | 0.38 | 0.729 | 14.93 | 68.50 | 7.46 |
|  | 0.50 | 0.736 | 13.71 | 66.50 | 6.71 |
|  | 1.00 | 0.750 | 13.31 | 62.29 | 6.22 |
|  | 1.68 | 0.721 | 13.53 | 60.02 | 5.85 |
|  | 2.10 | 0.735 | 13.13 | 59.96 | 5.79 |

**
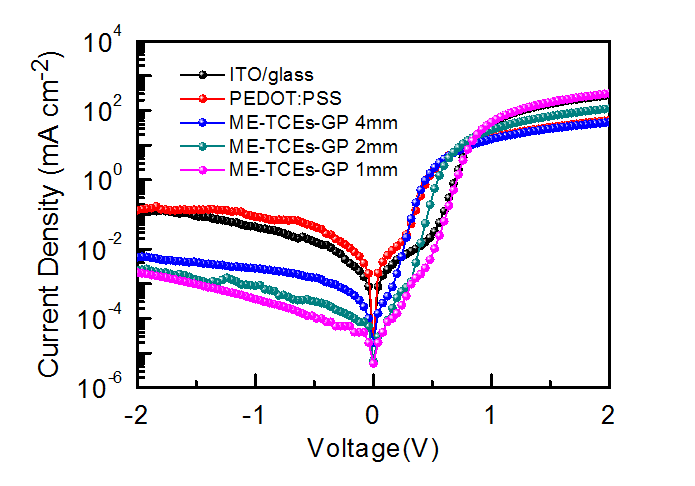
**

**Figure S3.** Dark *J*-*V* characteristics of IOSC device with various ME-TCEs GP sizes.


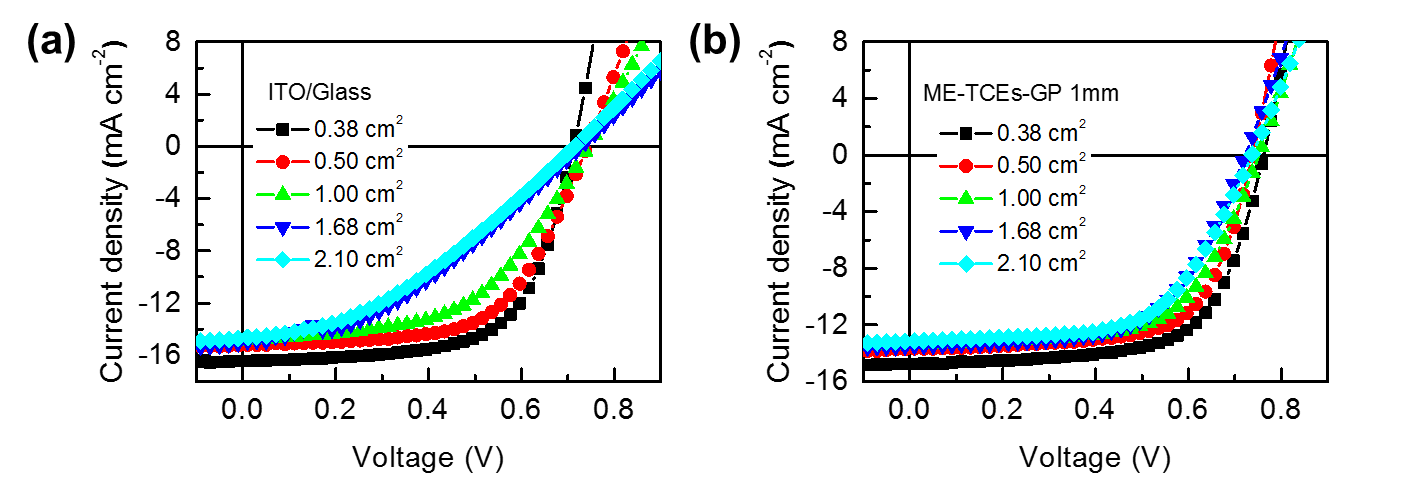
**Figure S4**. *J-V* characteristics of IOSCs based on (a) ITO/glass and (b) ME-TCEs-GP 1 mm substrates with various device active areas.


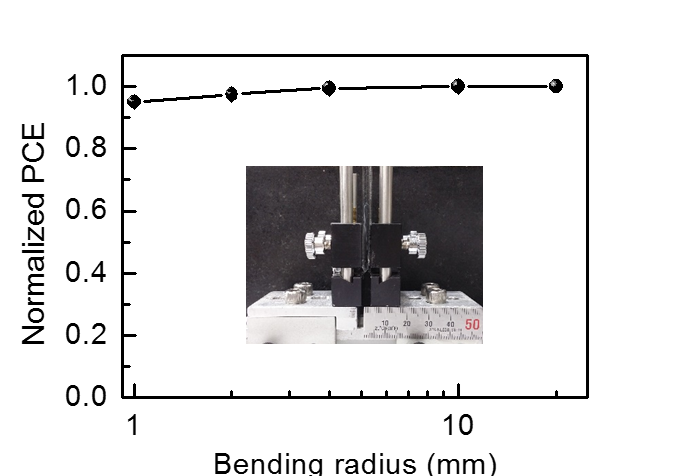


**Figure S5.** Normalized PCE values of IOSCs devices as a function of bending radii (ME-TCEs with a GP of 1 mm).

**Figure S6.** The *J-V* characteristics of flexible OLED devices.

**Supplementary Movie 1**

This movie shows a fabrication process of the metal grid-embedded transparent conducting electrodes (ME-TCEs).

**References**

[1] B. Friedel, P. E. Keivanidis, T. J. Brenner, A. Abrusci, C. R. McNeill, R. H. Friend and N. C. Greenham, Macromolecules, 2009**,** 42, 6741-6747.
